# Cephalopod inspired self-healing protein foams for oil-water separation

**DOI:** 10.1016/j.isci.2023.108300

**Published:** 2023-11-15

**Authors:** Khushank Singhal, Tarek Mazeed, Melik C. Demirel

**Affiliations:** 1 Department of Engineering Science and Mechanics, The Pennsylvania State University, University Park, PA 16802, USA; 2 Huck Institutes of Life Sciences, The Pennsylvania State University, University Park, PA 16802, USA; 3 Materials Research Institute, The Pennsylvania State University, University Park, PA 16802, USA

**Keywords:** Bioengineering, Materials science, Protein

## Abstract

Cephalopods are remarkable creatures, captivating scientists with their advanced neurophysiology, complex behavior, and miraculously effective camouflage. Research into cephalopods has led to many discoveries in neuroscience, cell biology, and materials science. Specifically, squids provide us with remarkable self-healing Squid Ring Teeth protein, which is applied herein to extend the life span of foams. Despite the advantages of porosity in surface science applications, porosity impairs mechanical properties by making materials more prone to structural damage –which traditional polymeric foams also suffer from. Drawing inspiration from Squid Ring Teeth, we developed self-healing tandem repeat proteins to overcome these challenges. By leveraging porosity and self-healing properties inspired by Squid Ring Teeth, we created bioengineered protein foams with high separation capacity (5.1 g g^−1^) and efficiency (≈94%). The foams healed entirely within minutes which regained over 100% strength after repair. These advances promise applications for efficient continuous water treatment through durable filter cartridges.

## Highlights

• Bioengineered foams with high separation capacity and efficiency are created
• Tandem Proteins can heal themselves, even in the smallest of pores
• Biomanufactured Squid Ring Teeth proteins are self-healing
• Sustainable Porous foams from recombinant proteins are produced

## Graphical abstract

**Figure:**
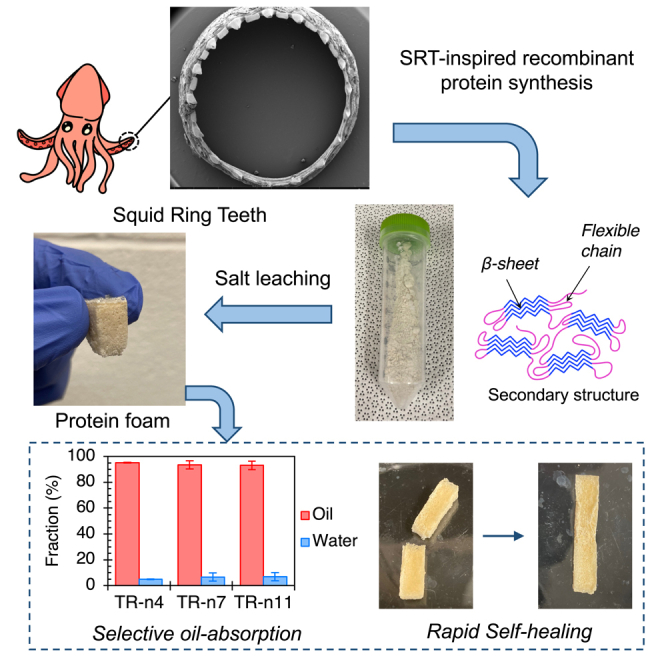

## Introduction

Self-healing is a remarkable natural phenomenon that allows organisms to repair internal or external damages and prolong their lives. The adaptive capabilities of organisms have enabled them to survive and thrive despite obstacles, such as external physical damage.^1^ Yet, at the cellular level, lies a prowess empowering life for millennia – self-healing. With this intrinsic and involuntary cellular process, living things can cope with their environment while maintaining sustainability. Scientists from many disciplines seek ways to harness this desirable capability by promising cost-effectiveness through longer product lifespans and greater environmental resilience.^2^^,^^3^ Despite significant attention toward developing self-healing polymers in recent years, many of these materials still suffer from low repair strength or slow healing times. However, diverse strategies to create advanced polymeric systems with enhanced qualities are still under development.^3^ While scientists have focused primarily on analyzing such creations with synthetic polymers, there is much room for innovation in bioengineered proteins that could revolutionize material engineering as we know it today.

Nature has an incredible ability to heal itself, even in the smallest of pores. We see this with bones and exoskeletons in animals and wood in plants - all have remarkable self-healing capabilities that surpass human engineering efforts.^4^ Hence, achieving self-healing in porous structures is challenging compared to bulk matrices. Yet, nature can still manage repairs without sacrificing the porous morphology. These fascinating capabilities of Nature motivated this research to create biosynthetic materials with similar resilience.

Porous materials are common in the industry, but their unique porosity can drastically reduce mechanical strength. This problem is further exacerbated by the random pore distributions that create stress concentrations that propagate failure upon an external load being applied. To counteract this effect, we looked to Nature for a self-healing material solution, namely squid ring teeth (SRT).^5^ Amazingly, SRTs contain pores - essential for our desired structure – and feature excellent healing capabilities without needing complex biological pathways. With SRT’s help, we could create repairable porous structures.^6^ We have achieved facility-scale production of bioengineered SRT proteins. These materials exhibit rapid self-healing and impressive strength recovery, repairing themselves in less than 1 min with tensile strengths ranging from 10 to 100 MPa.^7^ These proteins are classified as thermoplastic elastomers with hydrogen bonds that rapidly restructure protein chains at damaged interfaces into nanocrystal β-sheets, requiring only brief exposure to a plasticizer for activation.^8^

Here, we synthesized porous SRT protein foams using the salt-leaching method. Besides the rapid self-healing ability, we demonstrate how these bioengineered proteins can tune the mechanics of foams by their molecular weights. Additionally, SRT proteins' hydrophobic properties make them ideal for creating highly selective and efficient oil-absorbing foams. We show that the total oil absorption capacity and separation efficiency are 5.1 g g^−1^ and 94%, respectively. These self-healing foams are promising for several applications beyond solely structural, such as tissue scaffolding, acoustics, energy storage, and so forth. Combined with their wetting behavior, tandem repeat protein foams are an excellent choice as durable filter cartridges for water treatment. Moreover, our approach supports a sustainable circular economy, which can recover, reuse, and recycle protein foam materials.

## Results and discussions

### Tandem repeat proteins

**Figure 1:**
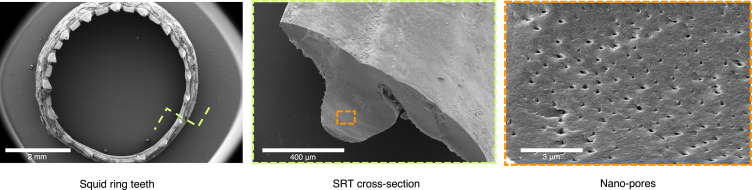
Scanning electron micrographs illustrating nano-pores in squid ring teeth cross-section at three different resolutions

Over a century ago, Williams' "Anatomy of Squid" described a principal evolutionary aspect of squid, i.e., teeth-containing suckers.^9^ Nixon and Dilly published an article in 1977 after studying squid suckers.^10^ They concluded that the suckers have a porous protein structure called squid ring teeth (SRT). Demirel et al. showed that SRT have self-healing abilities to repair themselves under pressure.^6^ We further studied the features of SRT and re-confirmed nanopores through scanning electron microscopy (SEM), as shown in Figure 1.

**Figure 2:**
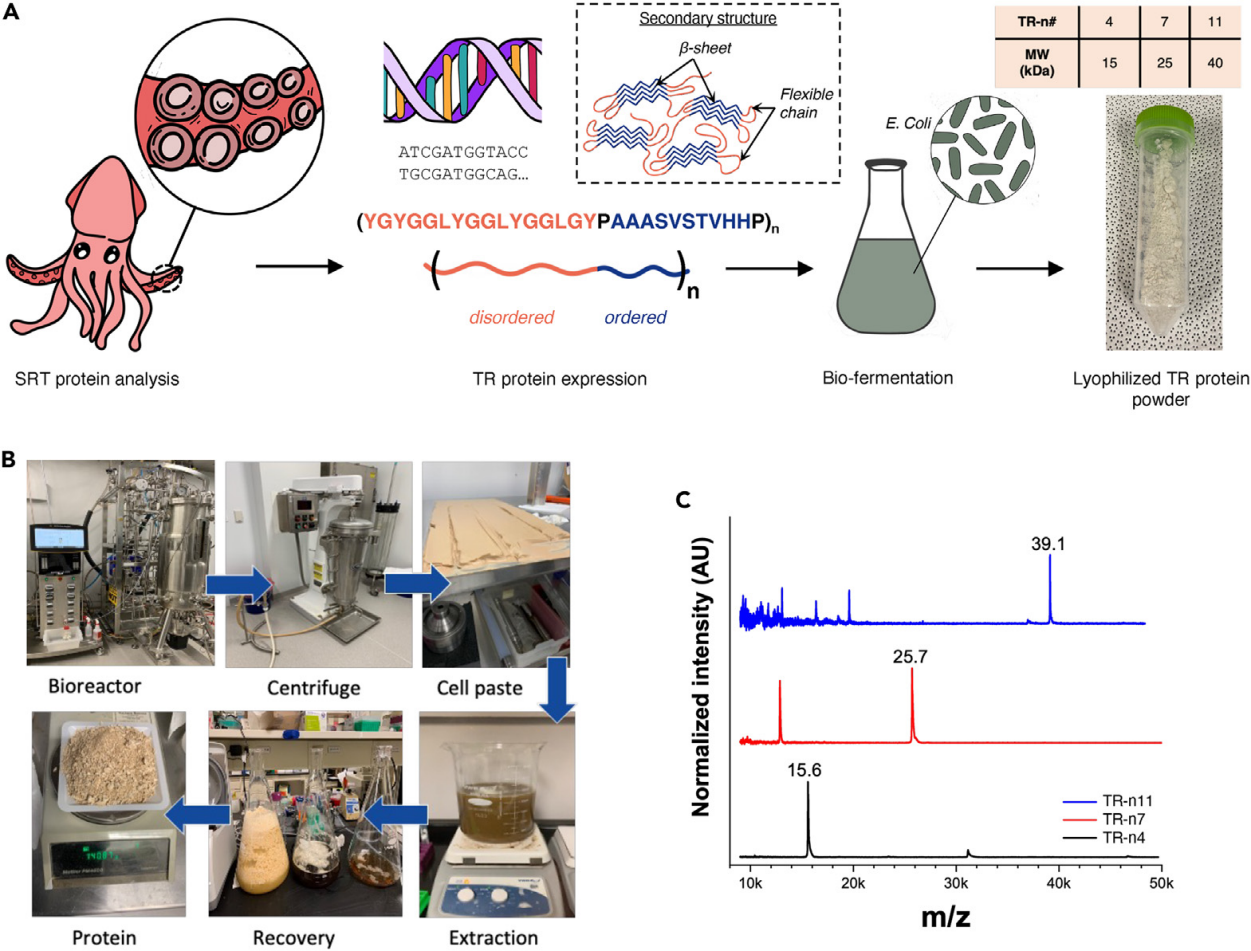
Biomimetic Tandem Repeat protein synthesis (A) Tandem repeat protein sequence design was inspired by squid ring teeth protein. Shown is the production process, including protein sequencing, expression in *E. Coli* followed by bio-fermentation, and downstream processes to yield protein powder. (B) Images showing production steps post-sequencing. (C) MALDI-TOF validation of molecular weight for purified TR proteins for TR-n4, n7, and n11.

Self-repairing materials can be engineered by manipulating protein structures at a molecular level. By varying amino acid sequences in tandem repeat (TR) proteins, inspired by the hierarchical assembly found naturally within SRT proteins, it is possible to design biomaterials with mechanically superior properties and self-healing capabilities. Specific alanine-rich segments (i.e., PAAASVSTVHHP) create physical crosslinks, while glycine regions (i.e., YGYGGLYGGLYGGLGYG) add flexibility to the polymer matrix making up these TR proteins. Furthermore, this segmented copolymer design approach enables us to adjust chain length, resulting in three distinct varieties: TR-n4, TR-n7, and TR-n11, where “n” denotes the number of repeat units. The resulting variations of 4-, 7-, and 11-repeat units were synthesized via microbial fermentation technique, as depicted in Figure 2A.

As reported in our earlier work, we used the E. coli strain BL21(DE3) to produce the TR proteins.^11^ Our organic extraction method enabled us to efficiently separate TR proteins, which are insoluble under cellular physiological conditions. By dissolving the inclusion body pellets in dimethyl sulfoxide (DMSO) and precipitating them with ultra-purified water as a counter solvent, we achieved a high yield of 1.5 g/L dry protein on both small and large scales (100 L fermenter). The process of synthesis is shown in Figure 2B. The molecular weight assessment was performed via mass spectroscopy (Matrix-Assisted Laser Ionization-Time of Flight spectroscopy, MALDI-TOF) because these proteins are insoluble in conventional buffers. More importantly, the protein electrophoresis gels are difficult to obtain and do not provide accurate results; MALDI-TOF provides accurate quantitative data (Figure 2C).

### Tandem repeat protein foams synthesis and structure

**Figure 3:**
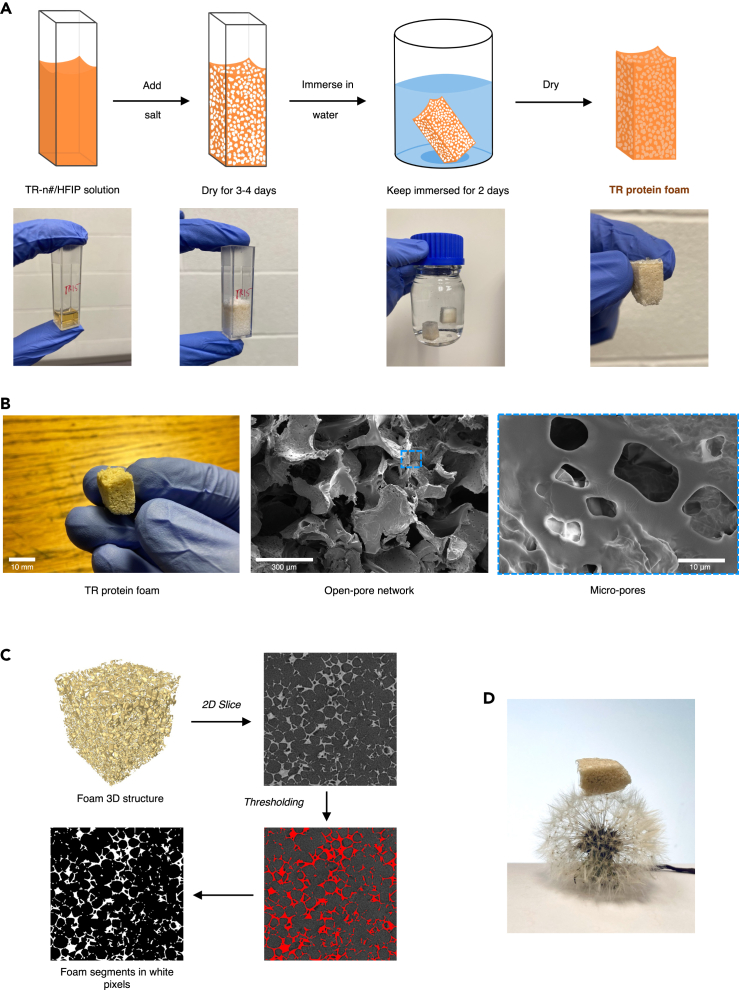
Morphology of TR protein foams (A.) TR protein foam synthesis by salt leaching method. (B) Interconnected pore network as seen in TR protein foam micrographs. The protein segments forming the foam structure further have micro-pores. (C) The process of estimation of foam porosity. Image analysis identifies protein segments in the 2D tomography scans. The fraction of black pixels in the final image represents porosity. (D) Ultralight TR protein foam supported by a Dandelion flower.

We prepared highly porous TR protein foams with open-cell morphology using the salt-leaching approach, as shown in Figure 3A. Our salt leaching process involves the use of an organic solvent and solid salt granules as porogen in an organic solvent (HFIP) that can dissolve our protein at high concentrations (i.e., >150 g/L). As HFIP evaporates, protein aggregates in the gaps between adjacent salt particles. Our study uses NaCl because it does not mix with the organic solvent (HFIP). To remove the salt, we utilize water, which eliminates the NaCl granules quickly but cannot dissolve the TR protein. We achieved an interconnected network of large pores formed upon the solidification of protein surrounding NaCl granules upon the evaporation of the volatile solvent. This method was chosen over gas-foaming as it yields homogeneous porosity distribution and an interconnected network of open cells throughout the material. Upon closer inspection through SEM (Figure 3B) and X-ray tomography (Figure 3C), the large pores encase smaller micro-pores, likely increasing fluid take up and saturation. The inter-spacing between NaCl crystals decides the thickness of the solidified protein segments and, thus, the formation of either the macro- or micro-pores. The calculated average porosities were 87.6 ± 1.8%, confirming the low densities of this bioengineered foam structure; a dandelion spore supporting a representative foam is shown (Figure 3D).

### Mechanical properties and self-healing

**Figure 4:**
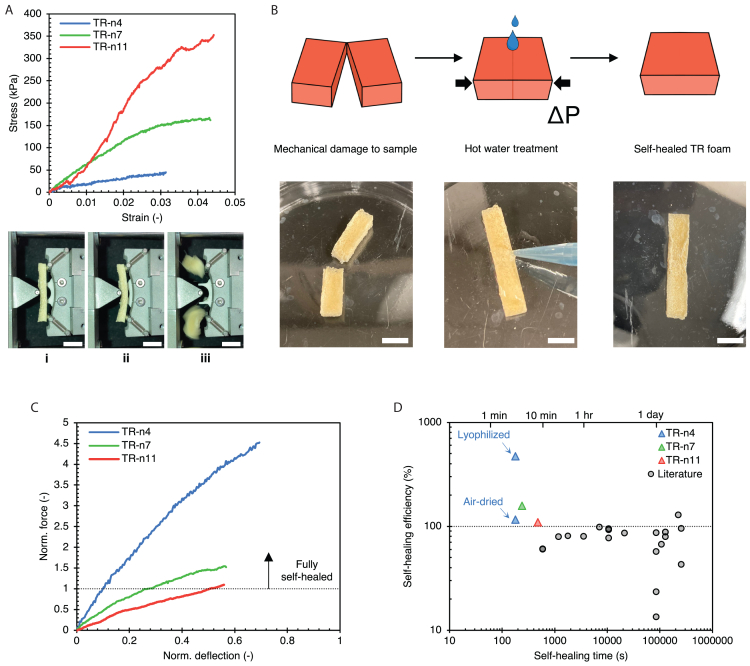
Mechanical and self-healing properties of TR protein foams (A) The strength and stiffness of pristine TR protein foams are tunable depending on the molecular weight of the protein chain. Representative stress-strain plots are shown, and a three-point bending test of a representative foam is depicted in chronological order (i, ii, and iii). Scale bars = 10 mm. (B) Description of self-healing protocol for foams after sustaining mechanical damage. Scale bars = 10 mm. (C) Norm. force-deflection curves of self-healed TR protein foams. The force-deflection curves of self-healed foams were normalized with respect to the max force and displacements of pristine foams. The data for TR-n4 represent lyophilized foams. The threshold line marks max flexural forces for the foams. (D) The figure-of-merit depicts the superior self-healing capabilities of TR protein foams as opposed to porous structures reported in the literature.^13^^,^^14^^,^^15^^,^^16^^,^^17^^,^^18^^,^^19^^,^^20^^,^^21^^,^^22^^,^^23^^,^^24^^,^^25^ While most studies used mechanical tests to validate self-healing, some used other functional characterization.

TR protein foams exhibit self-healing capabilities, which we validated through three-point flexural tests. The flexural stress-strain plots of the foams are depicted in Figure 4A, and the progression of a representative flexural test is shown in Figure 4A i, ii, and iii. As the molecular weight of TR protein increases, the ultimate stress and stiffness increase. The ultimate flexural stress for TR-n4, n7, and n11 are 62.2 ± 17.1 kPa, 167.2 ± 19.2 kPa, and 336.4 ± 23.8 kPa, resp. This behavior is expected because a higher degree of polymerization promotes chain entanglements and the formation of β-sheet structures. This favors the reduction of the density of network defects within the bulk of the protein matrix.^12^ The foams were self-healed after mechanical testing, and the regenerated strength was measured. After these rigorous experiments, a full-strength recovery after self-healing was confirmed.

Figure 4B showcases the self-healing protocol requiring no additional chemical treatment for repair. The mechanism of self-healing has been discussed earlier.^7^ Rapid chain diffusion occurs across fractured surfaces to form new secondary structures (e.g., β-sheets), speeding up the repair time. Hot water treatment lowers glass transition temperatures while external pressures (minimal in magnitude) ensure that the contact between damaged surfaces is maintained without compromising integrity. Figure 4C shows normalized deflection curves, confirming complete strength regeneration in reference to pristine foams (raw data are plotted in Figure S1). The strength regeneration was over 100% for all foams as indicated by max flexural force lying over the threshold (norm. force = 1) set by pristine foams. Additionally, the slopes of the initial linear region of force-deflection curves of self-healed foams were higher than that of pristine foams, indicating greater stiffness. A decrease in cross-sectional areas was expected during self-healing due to the shrinkage of soft protein matrices upon exposure to hot water. However, this decrease was significant only in the low molecular weight TR-n4 variant. We also analyzed a less porous TR-n4 foam that was air-dried instead of lyophilized to confirm complete damage reversal, as shown in Figure S1. This foam showed no shrinkage at the interface but still had over 100% strength regeneration. In our earlier report on self-healing TR protein films,^7^ we identified a shift of protein microstructure toward higher crystallinity (increased β-sheet fraction) due to hot water treatment. We posit that this increment in crystallinity at the self-healed interface is the reason behind improved mechanical properties.

Self-healing chemical-based protocols are not appropriate for oil-separating foams as they are effective with foams of lower porosities and closed-cell structures. Moreover, such methods do not conserve the porous morphology after repair. Traditional foams need conditioning (e.g., near-IR^13^ and sunlight^14^ irradiation), high-temperature and pressure annealing (vs. short treatment), or unique blends of chemicals, which take days to achieve self-healing. However, TR-n4, n7, and n11 foams can self-heal within 3–8 min. TR protein foams perform better self-healing than previously reported ones, as shown in Figure 4D. They can quickly and easily self-heal, allowing for the shaping of the foam as desired.

### Wetting properties of tandem repeat protein films

**Figure 5:**
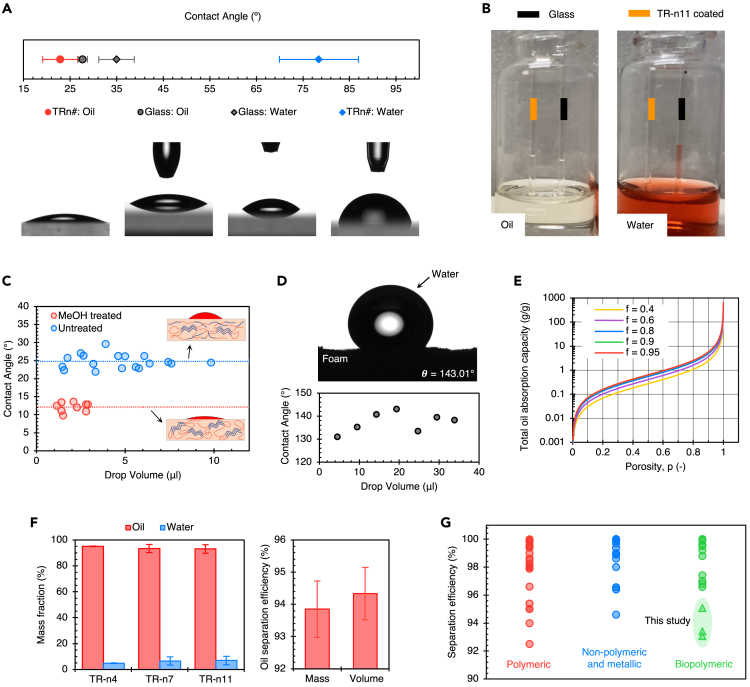
Wetting properties of TR proteins (A) TR proteins are hydrophobic and oleophilic compared to the glass substrate, as depicted in the plot and the contact angle images for respective cases. (B) Capillary rise experiment with glass and TR-n11 protein-coated capillaries with oil and water. The difference in column heights of liquids confirms TR proteins’ oleophilic and hydrophobic properties. (C) The contact angle of oil on washed and methanol-treated films. (D) The water droplet image on a representative TR protein foam sample and the respective plot of contact angle vs. droplet volume. (E) Theoretical estimate of total oil-absorption capacities (with 0.915 g cm^−3^ oil density and 1.35 g cm^−3^ material density) as a function of foam porosity at various selective oil-absorption efficiencies (f). (F) Fraction of oil and water absorbed by TR protein foams corroborates the selective oil absorption behavior. (G) Classification of foams reported in the literature (circles) according to the materials used and their selective oil-separation efficiencies.^37^^,^^38^^,^^39^^,^^40^^,^^41^^,^^42^^,^^43^^,^^44^^,^^45^^,^^46^^,^^47^^,^^48^^,^^49^^,^^50^^,^^51^^,^^52^^,^^53^^,^^54^^,^^55^^,^^56^^,^^57^^,^^58^^,^^59^^,^^60^^,^^61^^,^^62^^,^^63^^,^^64^^,^^65^^,^^66^^,^^67^^,^^68^^,^^69^^,^^70^^,^^71^^,^^72^^,^^73^^,^^74^^,^^75^^,^^76^^,^^77^^,^^78^^,^^79^^,^^80^^,^^81^^,^^82^^,^^83^^,^^84^^,^^85^^,^^86^^,^^87^

We conducted experiments to investigate how TR protein films interact with liquids, specifically water and oil, and how porosity affects these interactions. We measured the contact angles of both liquids on the films and found that water had an average contact angle of 78.43 ± 8.48°, while oil had an average contact angle of 22.86 ± 3.73°. The results indicate that TR proteins are not very hydrophilic but are highly oleophilic. Figure 5A shows each liquid’s corresponding average contact angles (see Supplemental information, Figure S2). TR proteins are more hydrophobic than natural biopolymers, including silk,^26^^,^^27^^,^^28^ zein,^29^^,^^30^ soy protein,^31^ keratin,^32^^,^^33^ and cellulose.^34^^,^^35^^,^^36^ We further demonstrate the oleophilic nature of TR proteins using the capillary rise experiment (Figure 5B). The difference in water column height between the protein-coated capillary and the control is over five times higher than the oil column. This selective-absorption behavior is crucial in foams, as will be discussed later. The wetting properties of the protein also depend on its degree of crystallinity. Thermal (hot water) and chemical (methanol) treatment of protein films induces higher crystallinity due to the formation of additional β-sheets. This can be quantified by the deconvolution of the Amide-I band of the FTIR spectra, as shown in Figure S3. The methanol-treated film had a higher fraction of β-sheet crystallites (39.97%) than the untreated film (30.13%). With the increase in crystallinity, the average contact angle of oil on TR-n11 decreased by 12.64° (as seen in Figure 5C). We note that the film could not be tested with higher oil droplet volumes due to the droplets' span exceeding the camera lens’s aperture (Figure S4).

### Selective oil-absorption

On porous foam surfaces, the contact angles of water and oil differ from those on thin films of proteins (Figure S5). Figure 5D depicts an image of the sessile drop experiment with water and TR protein foam. The contact angle of water rises to a maximum of 143.0 ° (with an average of 137.2 °), while the contact angle of oil could not be measured due to instant absorption. The experimental contact angle of water on foam is close to the theoretical estimate of 147.6 ° (see method details for derivation). These results demonstrate how porous surfaces amplify the hydrophobic behavior of TR proteins, which is comparable to other biopolymer aerogels^37^^,^^88^ of significantly higher porosities.

Numerous studies have been reported on oil-water separation in the literature.^89^ The most commonly used parameters to measure the performance of foams are total oil absorption capacity (g/g) and separation efficiency (the fraction of oil absorbed or separated). On average, the total oil-absorption capacity and separation efficiency are 5.07 ± 0.5 g g^−1^ and 93.9 ± 0.9%, respectively (Figure 5). The total absorption capacity can theoretically be estimated (Figure 5E), and the theoretical estimate for our foams (4.5 g/g) agrees with the experimental data. Our model indicates that it is necessary to normalize total oil-absorption capacity with porosity when using it as a figure of merit for wetting properties of porous materials (see method details and Figure S6). If fabricated with greater porosity, TR protein foams would exhibit high capacity (due to increased pore volume) while the protein’s wetting properties remain virtually unchanged. Hence, separation efficiency is a valuable metric for collectively comparing all porous materials' wetting behavior.

TR protein foams have a strong affinity for oil, which explains their selective oil-absorption behavior. Oil is quickly absorbed when the foam is placed at the liquid interface due to the capillary effect (see Figure S7). However, water is not absorbed because the surface is superhydrophobic. The material’s wetting characteristics and the liquid’s surface tension generate a pressure difference at the liquid-air interface within capillaries. This intrusion pressure promotes oil transport through capillaries while restricting water transport.^90^^,^^91^ When the contact angle inside capillaries is less than 90°, liquid transport is preferred, but not when it exceeds 90° (i.e., the lower the contact angle of liquid in capillaries, the faster the absorption kinetics). Our foams achieved saturation in under 40 s, indicating a fast oil absorption rate. Additionally, we observed that water did not displace the oil when an oil-soaked foam was immersed in water for extended periods.

In Figure 5G, we depict the separation efficiencies of different types of foams. This figure categorizes foams into four groups: polymeric, biopolymeric, non-polymeric, and metallic. Almost all foams mentioned in the literature are chemically modified. After the foam is produced, these modifications are done using agents such as silanes, siloxanes, metal oxides, and fluorinated polymers.^92^ The substrate is treated with these agents using dip-coating, chemical vapor deposition, polymer grafting, spray-coating, or nanoparticle deposition.^93^ On the other hand, our TR protein foams display high oleophilic behavior and comparable separation efficiencies even without any chemical enhancement of the surface. This highlights the uniqueness of TR protein foams as sustainable biomaterials, making them stand out from traditional sorbents. Figures S8 and S9 show the entire process flow of TR protein materials, highlighting their sustainability through reusability and recyclability after oil absorption.

## Discussion

Hydrophobic TR protein foams have unique properties that make them ideal for separating oil and water in water treatment processes. They can self-heal rapidly, a feature not reported in other open-celled foams. In addition to their use in industrial filtration processes, these foams are an excellent option for traditional porous sorbents of oil impurities. One of the benefits of TR proteins over synthetic polymeric systems is their sustainability. TR proteins are biodegradable and renewable, eliminating the need for petroleum-based everlasting polymers. The non-covalent nature of supramolecular interactions allows for easy repurposing of TR protein foams.

Biomanufactured TR proteins yield functional properties with high efficiency and sustainability. Remarkably, this replication of self-healing mechanisms allows for incorporating porosity into these proteins, thereby boosting their selectivity in oil absorption from aqueous media. This opens up exciting avenues, such as utilizing SRT foams to cleanse water or as efficient sorbents for oils without compromising performance due to deterioration.

### Limitations of the study

This study demonstrates that bioengineered protein foams, inspired by nature, can be sustainably utilized as absorbent materials. We showed that these foams have self-healing abilities to repair themselves under pressure. There is still a lack of understanding regarding the relationship between self-healing and porosity. Our research will focus on this topic in the future.

## STAR★Methods

### Key resources table

**Table:** 

REAGENT or RESOURCE	SOURCE	IDENTIFIER
**Bacterial and virus strains**		

E.coli BL21 strain	ATCC	469008

**Chemicals, peptides, and recombinant proteins**		

1,1,1,3,3,3-Hexafluoro-2-propanol	Oakwood chemicals	003409
Dimethyl sulfoxide	Oakwood chemicals	046777
Ultrapure water	Milli-Q	EQ 7000
Sodium Chloride	BDH	CAS: 7647-14-5
Pure canola oil	Great Value, Walmart	N/A
Isopropyl alcohol	VWR	BDH1174-4LP

### Resource availability

#### Lead contact

Further information and requests for resources should be directed to the lead contact, Melik C. Demirel (melik@psu.edu).

#### Materials availability

Experimental details can be found in the ‘method details’. The authors did not generate new unique reagents.

#### Data and code availability

• Authors declare that data supporting the findings of this study are available within the paper and its Supplemental information files. Raw data will be shared by the lead contact upon request.
• This study did not use a custom code deemed central to the conclusions.
• Any additional information required to reanalyze the data reported in this paper is available from the lead contact upon request.

### Experimental model and study participant details

#### Microbe strains

*E. coli* BL21(DE3) were inoculated and grown overnight in 5 mL LB with ampicillin (100 μg/mL), and grown in LB medium shaking at 37°C for 24 h.

### Method details

#### TR protein synthesis

TR proteins were engineered using protein expression, gene sequencing, and protein design according to a previously described protocol.^11^ The DNA sequences were verified in plasmids and then transferred to *E. coli* (BL21 strain with pet14b plasmid). After colony inoculation and fermentation, cells were collected and grown based on the earlier protocols. The fermentation biomass is processed to acquire purified TR proteins.

#### Downstream purification of protein

A unique approach based on Flory-Huggin’s theory was used to purify TR proteins with tandem repeat motifs. This approach involves using poor and good solvents to assemble the proteins into β-sheet, or α-helix stabilized aggregates. The purification process consists of two steps: dried cell paste is mixed with DMSO (up to 200 mg/ml) and spun down using centrifugation at room temperature. Second, a counter-solvent such as water, methanol, or ethanol precipitates the recombinant protein, which is then recovered from the pellets by decanting the liquid. This technique results in a high protein yield of over 80% due to increased hydrogen bonding of GLGY repeats in the amorphous region of the TR proteins. The solvent used in the separation and extraction steps of the purification is ranked by hydrogen propensity as DMSO > Water > Methanol > Ethanol.

#### Preparation of TR protein foams

TR protein foams were prepared using the salt-leaching method.^94^ TR-n#/HFIP solutions (#: 4, 7, 11) were prepared at a concentration of 15% (w/v) each. 0.75 ml solution was deposited in a cuvette, into which 2.25 g of granular NaCl was added slowly. The cuvette was covered with parafilm to achieve a homogenous distribution of salt within the solution and to prevent quick drying due to high solvent volatility. After settling of salt granules, parafilm was removed, and the protein-NaCl capsule was left to dry for 3 – 4 days. After that, the capsule was immersed in ultrapure water (for at least two days) for salt leaching. The water was changed several times to ensure complete salt removal. Finally, the resulting wet foam was allowed to dry completely.

#### Thin films for contact angle measurement

200 μl TR-n#/HFIP solution (1% w/v) was deposited on a glass slide and covered by a petri-dish to ensure homogenous distribution of solution on the substrate and smooth film surface due to slow drying (overnight). The glass slides carrying thin protein films were then immersed in ultrapure water for 30 minutes to wash out any traces of the solvent. The thin films were dried in the air and then transferred to a desiccator. Another film was treated with hot water (75°C) for one hr. and immersed in methanol overnight for secondary structure transition. The contact angle analysis was conducted on the Ramé-Hart Model 295 Automated Goniometer. The liquid, 1 μl or 5 μl in volume, was deposited on the film incrementally to obtain contact angles vs. droplet size.

#### Selective oil absorption

Ultrapure water and vegetable oil (5 ml each) were poured into a petri dish. A water-oil interface formed along the diameter of the petri dish (see Figure S7). Protein foams (of mass *m*_*f*_) were placed at the interface, ensuring contact with both liquids. The foams were removed after 1 minute and held in the air for 20 seconds to let excess liquid drop off. The liquid-soaked foams were then weighed (*m*_*f,o,w*_). They were kept in an oven at 65°C overnight to let the water evaporate and were measured subsequently (*m*_*f,o*_). The selective absorption efficiency of protein foams was calculated using the following equations:

Oil absorption (%) = 100×mf,o−mfmf,o,w−mf

#### Mechanical tests and self-healing

The protein foams were cast in molds of size 40 x 8 mm for three-point bending tests. The volume and concentration of the solution of each protein (with HFIP as solvent) were kept the same, i.e., 0.75 ml and 15% (w/v), respectively. All protein foams were of the same size. TR-n4 foams, instead of air-dried, were lyophilized. Pore collapse caused shrinkage during air drying due to their extraordinary softness when wet. The tests were conducted on a Psylotech μTS tensile tester at a deformation speed of 5 mm min^-1^. The fractured protein foams were self-healed. Hot water at ∼ 95°C was applied onto the broken region of the foams while ensuring contact between foam segments. After adequate water treatment, the foams were dried ambiently for two days before mechanical testing. Self-healing efficiency is the ratio of the maximum force of self-healed and pristine foams.

#### Other characterizations

SEM micrographs were taken using Quanta 250 ESEM and ThermoScientific Verios G4 UC FESEM. X-ray tomography of protein foams was carried out on the General Electric L300 system. The tomography scans were analyzed for porosity using ImageJ software. ATR-FTIR spectroscopy was carried out on Bruker Vertex 70 with a liquid nitrogen-cooled MCT detector using a Ge crystal accessory. A total of 256 scans collected at 3 cm^-1^ resolution were co-added. In addition, the capillary rise experiment was performed by coating the inner surfaces of glass capillaries (Kimble Chase, 1.5 – 1.8 mm) with TR protein solutions. 15 μl of TR-n#/HFIP solutions (concentration of 1% w/v) were deposited at one end of the capillaries using a micropipette and were allowed to travel through the length. The capillaries were dried in ambient conditions for two hours before immersing them in ultra-pure water. After drying, the capillaries were dipped in the desired liquid (water or oil), and the liquid menisci were noted at equilibrium. For recycling (Figure S8), the oil-soaked foams were treated with isopropyl alcohol (IPA) to extract the oil. After further washing with water, and drying, the protein foam was recovered efficiently. This recovered foam can either be re-used for oil absorption or recycled to fabricate new materials (like protein films). Fourier Transform Infrared (FTIR) spectroscopy of the recovered proteins revealed negligible oil content (Figure S9). This suggests that the affinity of TR proteins to bind with oil is low and they can be repurposed after oil separation efficiently. The recovered foam was dissolved in HFIP and drop-cast to make a regenerated TR protein film.

#### Theoretical models and calculations

The Cassie-Baxter model states that the apparent CA ($\theta^{\prime }$) of a liquid on a rough composite surface is^90^

(Equation 1) $$\cos\;\theta^{\prime }=f_{1}\;\cos\;\theta_{1}+f_{2}\;\cos\;\theta_{2}$$

where $f_{1}$ and $f_{2}$ are the fractions of the materials on the surface, and $\theta_{1}$ and $\theta_{2}$ are the Young’s CA of the liquid with the respective material. In our case, material 1 is either the liquid ($\theta_{1}=0{}^{\circ}$) or air ($\theta_{1}=180{}^{\circ}$), and material 2 is protein (see schematic in Figure S5).

TR proteins exhibit hydrophobic behaviour. Therefore, it is assumed that water does not penetrate the pores while wetting, and the two materials forming the composite rough surface are air and protein. From Equation 1,

(Equation 2) $$\cos\;\theta_{w}^{\prime }=0.87\;\cos\left(180{}^{\circ}\right)+0.13\;\cos\left(78.43{}^{\circ}\right)$$

$$\cos\;\theta_{w}^{\prime }=-0.87+0.026=-0.844$$

$$\theta_{w}^{\prime }=\cos^{-1}\left(-0.844\right)=147.56{}^{\circ}$$

we infer that the foams show superhydrophobic behavior. In case of oil-wetting, the air in the pores is replaced by oil, and oil and protein form the composite surface. Using Equation 1 again,

(Equation 3) $$\cos\;\theta_{o}^{\prime }=0.87\;\cos\left(0{}^{\circ}\right)+0.13\;\cos\left(22.86{}^{\circ}\right)$$

$$\cos\;\theta_{o}^{\prime }=0.87+0.119=0.989$$

$$\theta_{o}^{\prime }=\cos^{-1}\left(0.989\right)=8.51{}^{\circ}$$

we conclude that the foams are superoleophilic.

Here, we present a theoretical model based on pore saturation to understand the influence of various factors in liquid absorption by porous structures. The total oil absorption capacity (C) can be expressed as a function of porosity (p), material density (ρ), oil density ($\rho_{oil}$), and selective oil-absorption efficiency (f = volume of oil/total volume of liquid absorbed) as follows,

(Equation 4) $$C=\frac{pf\rho_{oil}}{\left(1-p\right)\rho }$$

assuming negligible material swelling. Considering the logarithmic trend of the total oil-absorption capacity vs. porosity, it is clear that total oil-absorption capacity is highly contingent on porosity. Moreover, the effect of material bulk density on total oil-absorption capacity is not as significant (see Figure S6B). While the material properties do overpower p, their effect is visible only at the extremities. Therefore, for a fair comparison of wetting properties of porous materials, we should employ separation efficiency. This is also beneficial from the viewpoint of purity of the filtrate in many applications. Porous materials that can ensure extremely low contamination of the filtrate (e.g., cleaning oil microdroplets from water) are more desirable.

### Quantification and statistical analysis

No statistical analysis is used in the study.
