# Supplementary material for: Cephalopod inspired self-healing protein foams for oil-water separation
Source: iScience. 2023 Nov 15;26(12):108300. doi: 10.1016/j.isci.2023.108300 (PMC10767161; doi:10.1016/j.isci.2023.108300)
Supplement: Document S1. Figures S1–S9 [file mmc1.pdf]

iScience, Volume *26*

Supplemental information

Cephalopod inspired self-healing

protein foams for oil-water separation

Khushank Singhal, Tarek Mazeed, and Melik C. Demirel

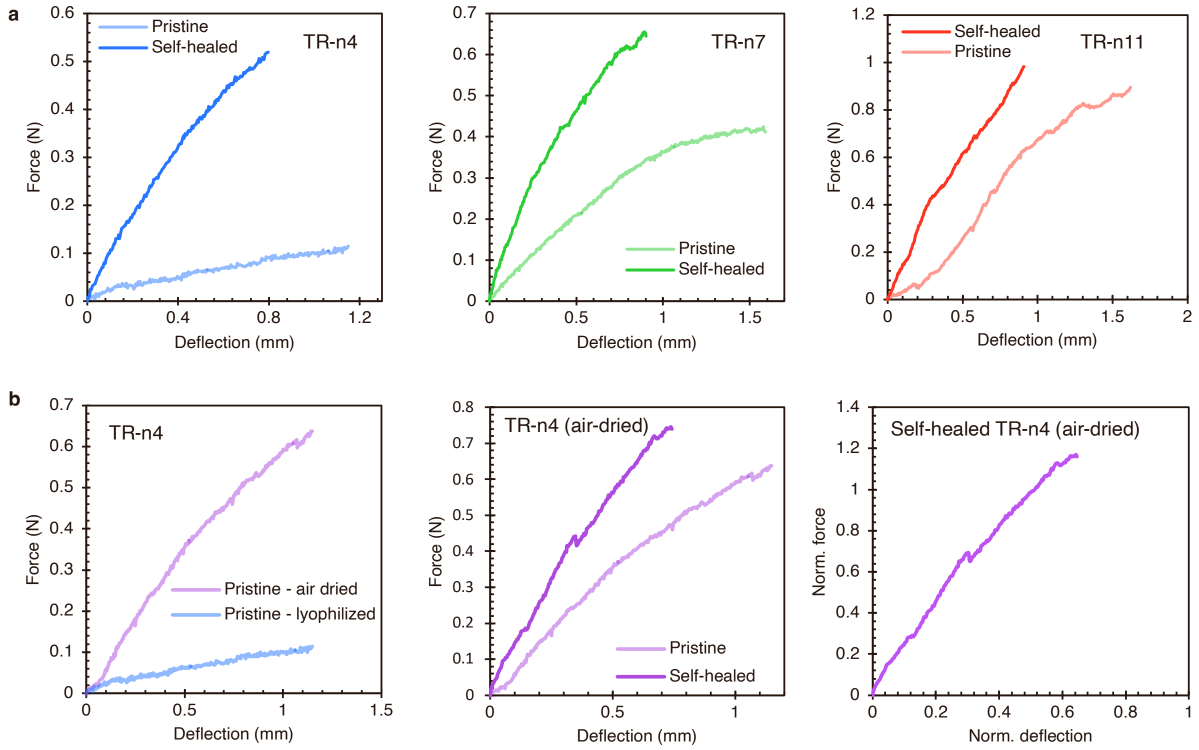

Figure S1. Three-point bending tests of TR protein foams, related to Figure 4.

a). Force-deflection curves for self-healed and pristine foams.

b). Force-deflection curves for TR-n4 foams illustrating the influence of size on mechanical properties. The ultimate breaking force and stiffness of air-dried foams were considerably higher than that of lyophilized foams. This was due to the higher density of air-dried foams. The average thickness of air-dried foams was 2.84 mm, whereas, for lyophilized foams it was 4.1 mm. The strength regeneration upon self-healing in the case of air-dried foams was smaller than lyophilized foams. This is so because air-dried TR-n4 foams were compact to begin with and the self-healing process did not cause any significant change in density (as the foam dimensions remain unchanged).

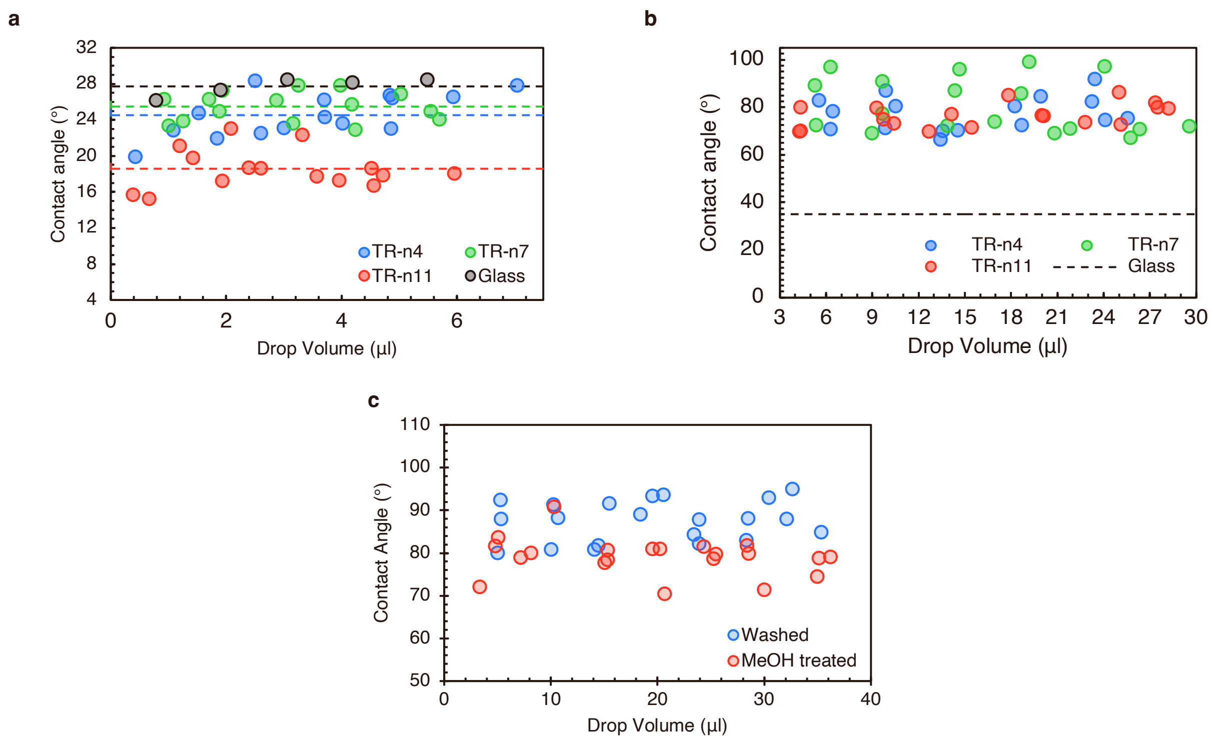

Figure S2. Sessile drop experiments with TR protein films, related to Figure 5a and 5c.

a). Contact angles of oil on TR protein films with respect to drop volume. Despite the proteins being varying in molecular weights, no significant trend of contact angle vs. molecular weight was noticed – only that TR-n11 is most oleophilic on average.

b). Contact angles of water on TR protein films with respect to drop volume. We postulate these overlapping distributions to have arisen mainly due to the droplet pinning effect. When a droplet is not allowed to spread (i.e., the contact line is pinned on the surface), a contact angle greater than which is real is observed. The pinning effect was more pronounced in the case of water.

c). Contact angle of water on methanol-treated and a pristine TR protein film. With the increase in hydrophobicity, the adhesion force should reduce, and thus, pinning as well. This can be noticed with methanol treated film, wherein, the average contact angle of water is lower than that of the un-treated film.

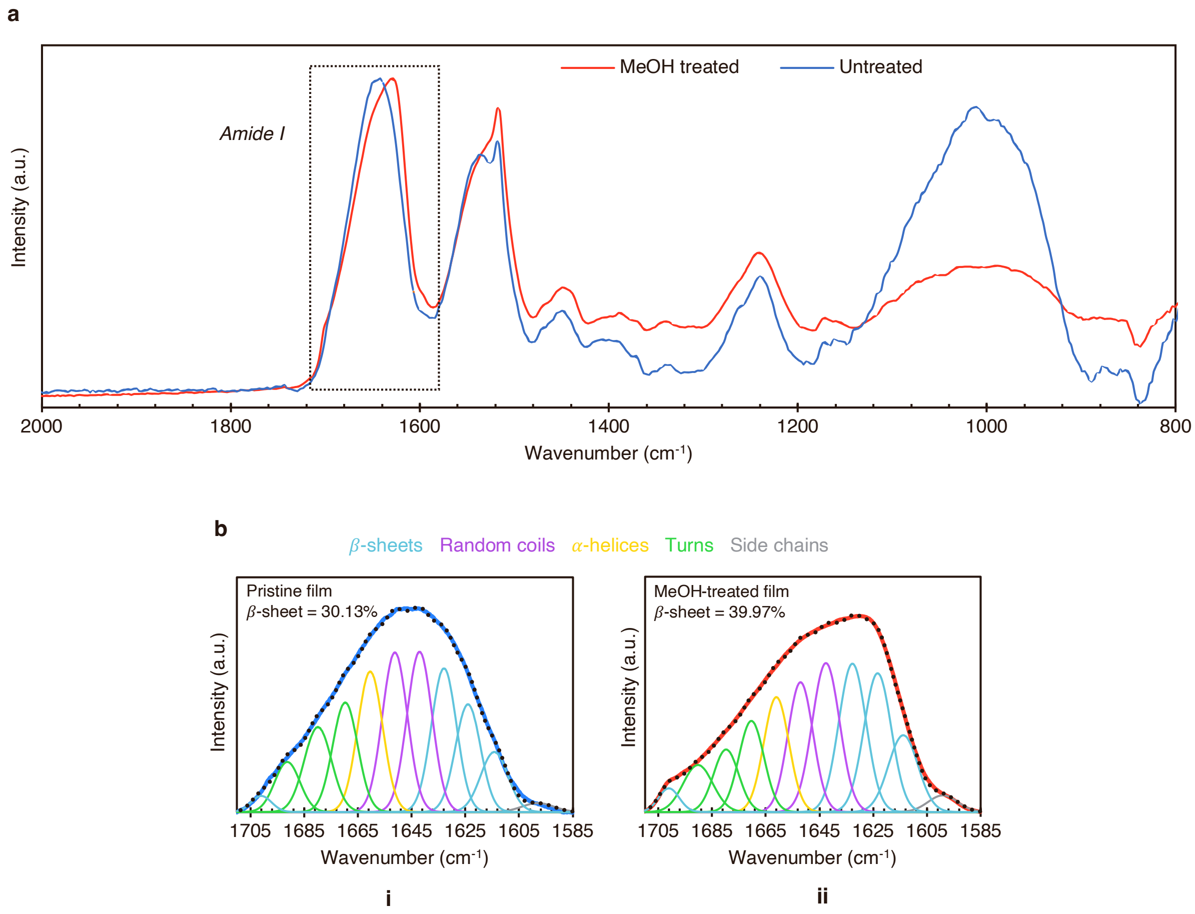

Figure S3. ATR-FTIR spectra of protein films, related to Figure 5c.

a). ATR-FTIR spectra of an untreated (pristine), and methanol treated film. Hot-water and subsequent methanol treatment induced beta-sheet structures in the protein film. The shift of peak-center of the Amide-I region towards smaller wavenumbers verifies this.

b). The deconvoluted and peak-fitted Amide-I bands of the original spectra of the two films. The deconvoluted spectra revealed the fractions of each of the secondary structures in the protein film, highlighting that the beta-sheet content increased by about 10% upon methanol treatment.

a

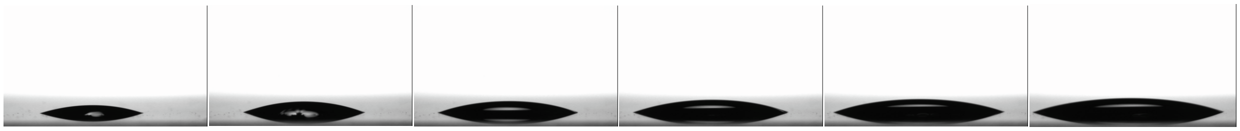

Evolution of contact angle of oil on untreated film with increasing droplet size

b

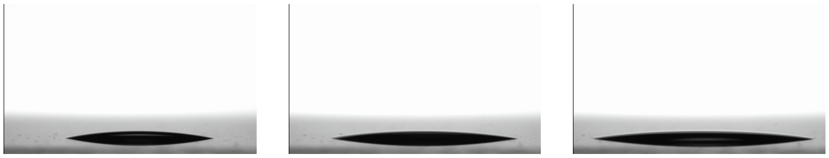

Evolution of contact angle of oil on MeOH-treated film with increasing droplet size

Figure S4. Optical images of the sessile drop experiments on a representative TR protein film, related to Figure 5c.

a). Untreated, or pristine, TR protein film with oil. The droplet contact line was observed to expand post liquid dropping and become stable in seconds. Such a feature was not seen with water. This phenomenon along with lesser statistical variation in contact angle indicates that the droplet pinning effect was significantly reduced with oil.

b). Methanol and hot water treated TR protein film with oil. The increase in crystallinity upon methanol-treatment induced a precise reduction in contact angle of oil on the film.

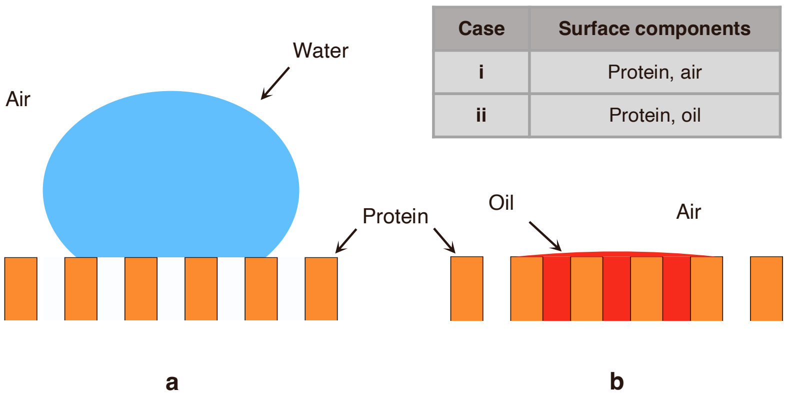

Figure S5. Schematic representation of the Cassie-Baxter wetting regime, related to Figure 5d.

a). When the test liquid does not penetrate the pores, the porous material and air form the composite rough surface.

b). When the test liquid penetrates the pores, the air is replaced by the liquid to form the composite rough surface.

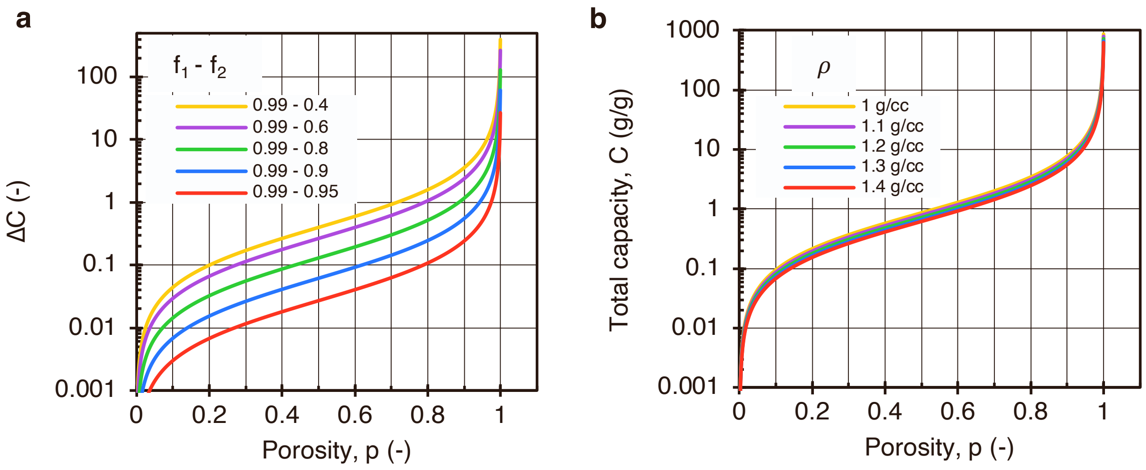

Figure S6. Theoretical trends of absorption, related to Figure 5e.

a). The difference in total oil absorption capacity vs. foam porosity for several combinations of selective absorption efficiency (for bulk material density = 1.35 g·cm^{-3}).

b). Total capacity vs. foam porosity for materials with varying bulk densities. Oil density was considered to be 0.915 g·cm^{-3}.

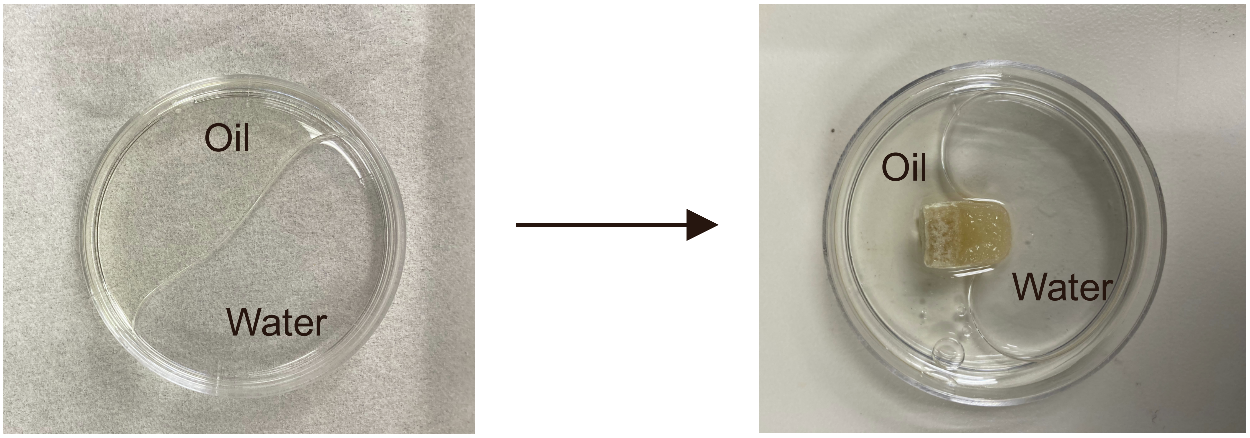

Figure S7. The experimental setup for measurement of the selective oil-absorption by TR protein foams, related to Figure 5f.

The oil and water separate along the diameter of the petri dish, and the foam was placed at the interface. Our protocol ensures contact of the foam with both test liquids simultaneously. Such arrangement is not done in studies reported in literature.

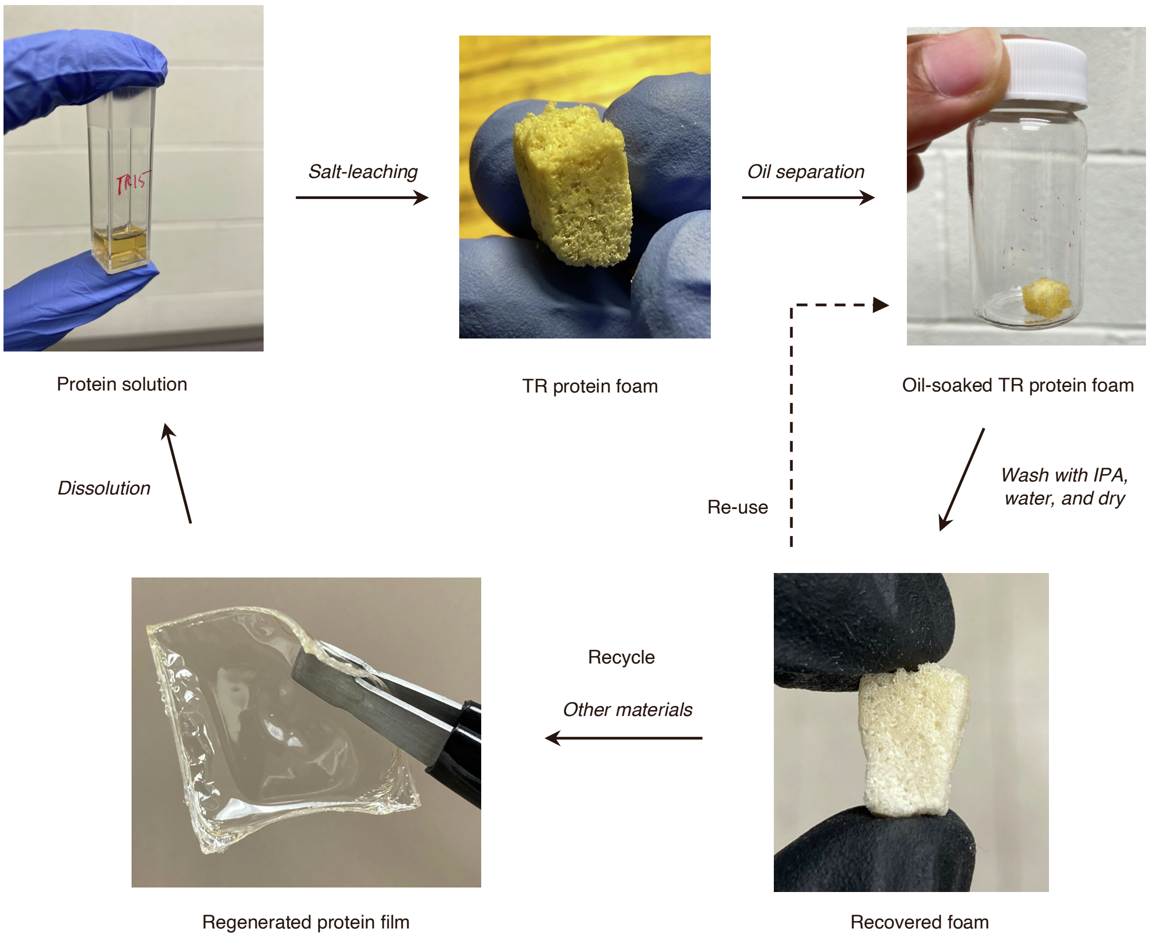

Figure S8. The lifecycle of TR protein foams and materials illustrating circularity of their use, related to Method Details.

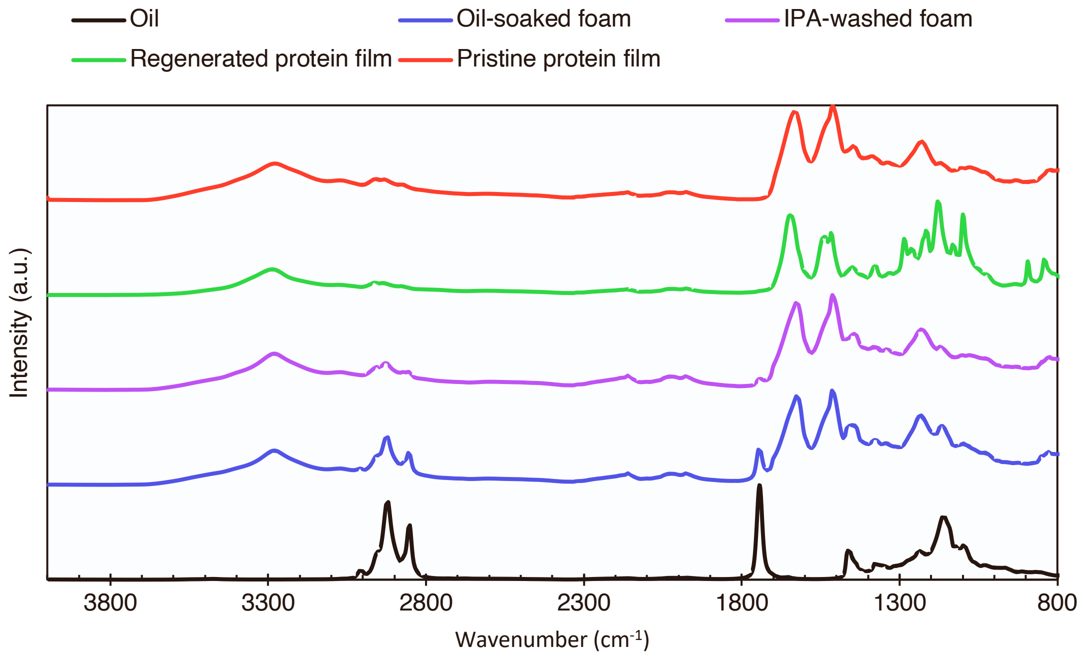

Figure S9. ATR-FTIR spectroscopy of a representative TR protein foam and associated materials along their lifecycle, related to Method Details.

The characteristic peak (ester) of oil appears at 1743 cm^{-1}. The ester peak in IPA-washed and recovered foam is negligible as compared to that in the spectrum the oil-soaked foam. It should be noted that the ester peak in the film-spectrum is even weaker than that of the recovered foam. This probably is due to incorporation of the remaining oil traces in recovered foam into the protein matrix during dissolution. This supports that the TR protein does not swell in oil, rather interacts with oil on the surface only.
